# A qualitative study on the feasibility and acceptability of institutionalizing health technology assessment in Malawi

**DOI:** 10.1186/s12913-023-09276-z

**Published:** 2023-04-11

**Authors:** Joseph Mfutso-Bengo, Faless Jeremiah, Florence Kasende-Chinguwo, Wingston Ng’ambi, Nthanda Nkungula, Isabel Kazanga-Chiumia, Mercy Juma, Marlen Chawani, Jobiba Chinkhumba, Pakwanja Twea, Emily Chirwa, Kate Langwe, Gerald Manthalu, Lucky Gift Ngwira, Dominic Nkhoma, Tim Colbourn, Paul Revill, Mark Sculpher

**Affiliations:** 1 Health Economics and Policy Unit, Department of Health Systems and Policy, Kamuzu University of Health Sciences (KuHes), Private Bag 360, BLANTYRE 3, Lilongwe, Malawi; 2 Centre of Bioethics in Eastern and Southern Africa, Blantyre, Malawi; 3 Centre of Excellence in Ethics and Governance, Blantyre, Malawi; 4 Malawi-Liverpool-Wellcome Programme, Blantyre, Malawi; 5 Ministry of Health, Malawi Government, Lilongwe, Malawi; 6 University College London, London, United Kingdom; 7 Centre for Health Economics, University of York, York, UK

**Keywords:** Health technology assessment, Health technology, Decision making, Institutionalization, Sub-Saharan Africa, Malawi

## Abstract

**Objective:**

The objective of this study was to assess the feasibility and acceptability of institutionalizing Health Technology Assessment (HTA) in Malawi.

**Methods:**

This study employed a document review and qualitative research methods, to understand the status of HTA in Malawi. This was complemented by a review of the status and nature of HTA institutionalization in selected countries.Qualitative research employed a Focus Group Discussion (FGD ) with 7 participants, and Key Informant Interviews (KIIs) with12 informants selected based on their knowledge and expertise in policy processes related to HTA in Malawi.Data extracted from the literature was organized in Microsoft Excel, categorized according to thematic areas and analyzed using a literature review framework. Qualitative data from KIIs and the FGD was analyzed using a thematic content analysis approach.

**Results:**

Some HTA processes exist and are executed through three structures namely: Ministry of Health Senior Management Team, Technical Working Groups, and Pharmacy and Medicines Regulatory Authority (PMRA) with varyingdegrees of effectiveness.The main limitations of current HTA mechanisms include limited evidence use, lack of a standardized framework for technology adoption, donor pressure, lack of resources for the HTA process and technology acquisition, laws and practices that undermine cost-effectiveness considerations. KII and FGD results showed overwhelming demand for strengthening HTA in Malawi, with a stronger preference for strengthening coordination and capacity of existing entities and structures.

**Conclusion:**

The study has shown that HTA institutionalization is acceptable and feasible in Malawi. However, the current committee based processes are suboptimal to improve efficiency due to lack of a structured framework. A structured HTA framework has the potential to improve processes in pharmaceuticals and medical technologies decision-making.In the short to medium term, HTA capacity building should focus on generating demand and increasing capacity in cost-effectiveness assessments. Country-specific assessments should precede HTA institutionalization as well as recommendations for new technology adoptions.

**Supplementary Information:**

The online version contains supplementary material available at 10.1186/s12913-023-09276-z.

## Introduction

Health systems in low-income countries (LICs), such as Malawi, are characterized by low per capita health spending. Limited budgets for effective healthcare provision have created a dire need for stronger resource allocation and prioritization processes as opportunity costs in sub-optimal decision-making lead to significant losses in the population’s overall health gains [1]. Globally, evidence shows that LICs face systematic challenges in optimizing already scarce resources in their health sectors. These challenges, therefore, undermine service delivery and the provision of equitable and quality health care that could easily be remedied through increased and efficiently allocated healthcare finances [2]. Thus, the pursuit of a strategy of evidence-based and optimal value-based decision-making frameworks through tools such as Health Technology Assessment (HTA) can help address issues relating to both affordability and equity in priority settings when allocating extremely scarce resources in these contexts [3, 4].

Health technology is defined as a health intervention that seeks to improve health and health sector outcomes by “preventing, diagnosing and treating medical conditions, promoting health or organizing health delivery” [5]. HTA is subsequently defined as “the systematic evaluation of the properties and effects of a health technology aimed at addressing the direct and intended effects of that health technology, as well as its indirect and unintended consequences’’ [5]. HTA is largely propagated in areas that target efficient and cost-effective health resource allocation, and in the prioritization, application and adoption of varied health technologies [5]. As a result, the widespread use of HTA holds prominence in medical, organizational, economic, ethical and societal means for technology adoption for a nation’s health sector at all levels of development [6].

HTA institutionalization is predominant in the context of high- and middle-income countries like the United Kingdom (through the National Institute for Health and Care Excellence (NICE) structure), Australia, Thailand and several European countries. In these contexts, HTA has a pivotal role in health technology prioritizationand decision-making. This leads to quality healthcare intervention adoption for their respective populations [2]. On the other hand, decision-making structures in many LICs like Malawi lack institutionalization, resulting in the inefficient and cost-ineffective allocation of scarce healthcare resources [3, 4]. Therefore, this study assesses the feasibility and acceptability of implementing an institutionalized HTA mechanism for the Malawian healthcare sector.

### Conceptual framework for institutionalization of HTA

“Institutionalizing” refers to the establishment of governance structures and pathways appropriate to yield technology assessments that are fundamental in guiding policy and clinical practice toward the best possible health and cost outcomes [6]. The World Health Organization (WHO) endorsed HTA as a tool to support open, “*evidence-informed priority setting, through systematic evaluation of the properties and effects of a health technology*” [7]. The use of HTA in LMICs is further advocated to determine the value of a health technology (e.g., a drug, medical device, diagnostic test, medical procedure) at different points in its lifecycle to inform the decision making to promote an efficient, equitable, and high-quality health system [5].

**Diagram 1:**
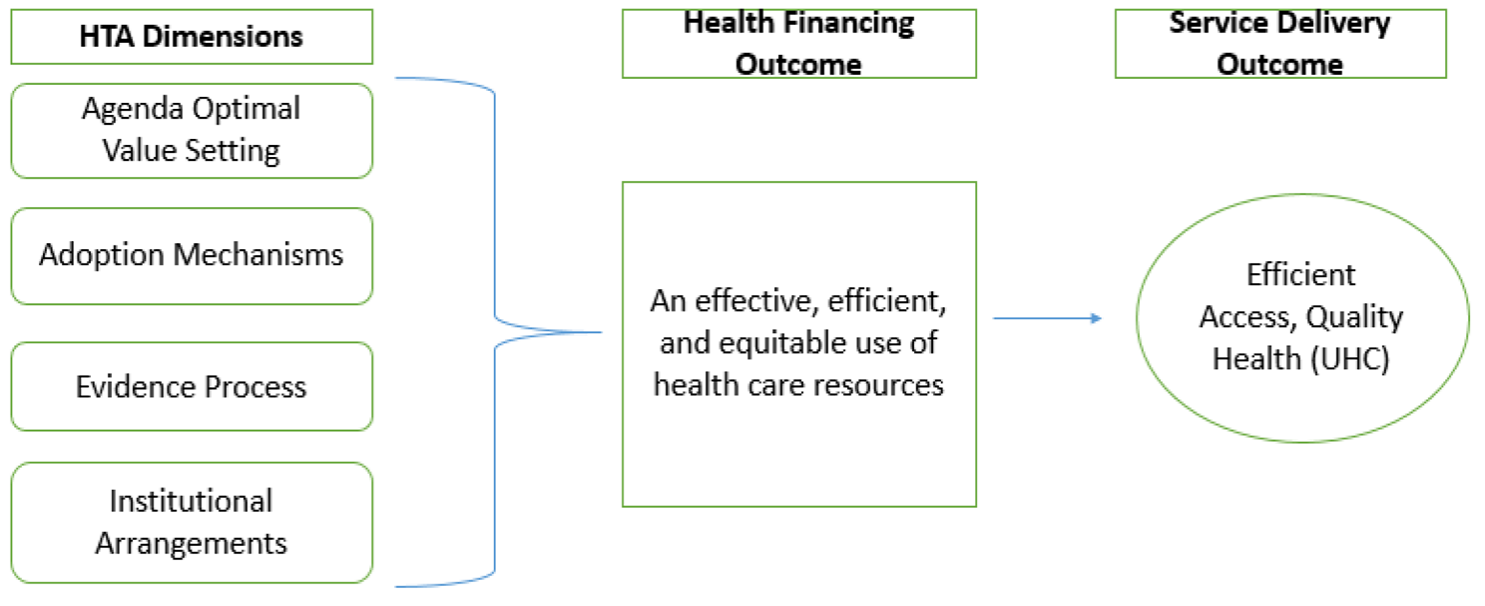
Conceptual Framework for HTA institutionalization *Source: Authors*

For a country to effectively achieve priority setting, it needs an institutionalized HTA. The tenets behind the successful institutionalizing of HTA as advocated by WHO is establishing the demand for HTA; institutional arrangements; a legal framework, Human resources capacity for HTA, HTA financing, HTA evidence and evaluation of the HTA mechanism [5]. Institutionalizing HTA promotes structures and processes suitable to produce technology assessments that are optimal in guiding policy and clinical practice toward the best health and cost outcomes [8]. The aim is to use HTA among relevant stakeholders to address health sector allocation challenges closing the knowledge gaps among HTA stakeholders and users directed towards improved and efficient service delivery outcomes [9]. *Diagram* 1 depicts the conceptual framework for institutionalizing HTA in a health care system, where the premise lies; HTA dimensions of agenda setting, having appropriate values for decision making, including robust evidence processes and institutional arrangements will thus lead to efficient and effective use of health care resources thus contributing towards the goal of UHC through efficient access to health resources [10]. There are no standardized models or universal paths for the development and institutionalization of agencies but rather considerations may be established based on a country’s cultures and values, healthcare systems, political priorities and governance.

## Methods

### Study design

This was an exploratory phenomenological qualitative study that employed a document review and qualitative inquiries through Focus Group Discussion (FGD) and Key Informant Interviews (KIIs). Data was collected between November 2020 and March 2021. Due to COVID-19 restrictions, interviews and the FGD were conducted primarily via phone call andzoom except for four face-to-face interviews that were conducted while adhering to COVID-19 guidelines.

### Data collection methods

#### Document review

An extensive literature review was conducted on relevant key policy documents, reports and publications on HTA in Malawi and selected countries. The aim of the literature review was, firstly, to understand the status of HTA in Malawi, and secondly, to conduct an environmental scan of HTA structures in various countries to identify HTA themes, values and institutionalization approaches in different contexts. Key relevant documents were identified through consultation with key informants from the Ministry of Health and an online literature search. PubMed, Cochrane, BMC and Science Direct were among the databases searched for retrieving literature for other countries. Key terms such as “health technology assessment”, “Biomedical Technology Assessment”, “Technology Assessment, Health”, “Assessment, Health Technology”, “Assessments, Health Technology”, “Health Technology Assessments”, “Technology Assessments, Health”, “Assessment, Biomedical Technology”, “Assessments, Biomedical Technology”, “Biomedical Technology Assessments”, “Technology Assessments, Biomedical”, “Technology Assessment”, “Assessment, Technology”, “Assessments, Technology”, “Technology Assessments”, “HTA values”, “structure of HTA in (country)”, “Southern African HTA Association” were used to identify relevant articles. The search was limited to articles published after the year 2010 and written in English and excluded articles whose emphasis was not on the data collection tool. We also consulted the websites of the following institutions: International Network of Agencies for Health Technology Assessment (INAHTA); Health Technology Assessment International (HTAi); European Network for Health Technology Assessment (EUnetHTA); NICE, United Kingdom; Agency for Healthcare Research and Quality (AHRQ), United States; Institute for Quality and Efficiency in Health Care (IQWiG),Germany; Haute Autorité de Santé (HAS), France; National Committee for Health Technology Incorporation (CONITEC), Brazil; and the Institute of Health Technology Assessment (IETS), Colombia.

#### Qualitative inquiry using FGD and KIIs

We conducted one FGD with 7 policy makers and 12 KIIs with key stakeholders. Thus, in total the whole qualitative sample constituted 19 participants and was based on data saturation whereby the information collected became repetitive and the collection of new data did not shed any further light on the issue under investigation. The participants were purposively selected and they includedofficials from the Ministry of Health (Headquarters, Central Hospitals, District Hospitals), Civil Society, Central Medical Stores Trust (CMST), Christian Health Association of Malawi (CHAM) and regulatory authoritiesi.e., Medical Council and Pharmacy and Medicines Regulatory Authority (PMRA). The FGD was used to gain an understanding of the decision-making processes related to health technology adoption in Malawi whilst the KIIswas used to gauge further individual perceptions, beliefs, and attitudes on identifying, prioritization, adopting and implementation of health technologies in Malawi.

### Data management and analysis

Data extracted from the literature was organized in Microsoft Excel and categorized according to thematic areas and analyzed using a literature review framework. The framework was used to capture the key relevant information and to synthesize it. All interviews and discussions were audio-recorded with unique identification assigned to each recorded interview. To enhance credibility, field notes were captured during data collection as well as triangulation discussions among researchers. All interviews were conducted in English as per the participants’ preference. The audio data was transcribed verbatim. Data analysis was conducted using a thematic analysis approach. Systematic coding was done and a code book in Microsoft Excel was used to organize and manage the data. Two researchers independently coded separate transcripts to assess the applicability of codes and any differences were resolved through iterative discussions between them. These were then validated by a third researcher. The analysis was done in five stages. The first stage involved going through all the transcripts to get familiar with the data. During this stage, short notes were made on transcripts and highlighter colors were used to indicate keywords or statements within the text. The second stage involved eliciting codes from text ‘line-by-line’ to generate initial codes. The third stage involved sorting and categorical grouping of codes into sub-themes. The fourth stage involved theme generation through the merging of sub-themes. The last stage involved producing the analysis report.

### Ethical consideration

The study was approved by the College of Medicine Research Committee (COMREC) in Blantyre, Malawi (protocol #: P.10/19/2820). Since this was not a clinical trial, we conducted the study in accordance with the COMREC guidelines and regulations for a non-clinical trial. We obtained informed consent from all the participants involved in the FGD. To protect the confidentiality of the participants’ information, no identifying information was included in the interview transcripts or FGD. The recordings and transcripts were kept in a password-protected computer with access limited to the researchers.

## Results

### Environmental scan HTA Institutions: World Health Organization (WHO), United Kingdom, Thailand, Ghana, Ethiopia and South Africa

The institutional contexts of HTA agencies are highly variable, presenting differences in the organization of the HTA process “chain of assessment” stages (prioritization, evaluation, appreciation, dissemination, and implementation of results/recommendations) [7]. The literature review aimed to conduct an environmental scan to retrieve sample HTA structures in various countries to understand various HTA themes and values and how institutionalization is conducted in varying contexts [8]. The diversity of the organization of HTA in different countries with regard to mission, structure, financing, priority setting and impact on decision-making was assessed using examples of known HTA agencies. While the focus was the WHO guidelines for effective HTA institutionalization, the UK NICE, Thailand, Ghana, Ethiopia,and South Africa structures also informed this assessment.

#### WHO

The WHO has been a key player in endorsing HTA as a critical tool in evaluation mechanisms. The WHO proffers that successful HTA programs require an appropriate education and training strategy targeted at expertise, organization and staff qualification. It postulates that the varying sectors of health technology systems, organizations, processes, procedures, devices and drugs should be subject to assessment, comparison and continuous improvement [9]. The WHO highlights generic areas such as, *“a focus on clinical databases on low-cost common technologies versus high technology; a correlation between output and content; bottom-up/top-down synergy; driven by professions not by industry; increased equity and access to emerging technology may be especially helpful in this regard”* [9].

#### UK

As an independent institution, NICE provides a roadmap in which quality health can be achieved in the United Kingdom [11]. NICE is responsible for appraising technology directed at clinical and cost-effectiveness of new or existing medicines and treatments and then providing corresponding feedback to the National Health Service [10]. NICE has a major focus on technologies that are in the realm of pharmaceuticals, devices, diagnostics, procedures and health promotion mechanisms [11]. The Key principles (normative values) of NICE guidance development are validating processes that include focused questions, stakeholder input, independent robust evidence assessment, independent advisory committee, multiple perspectives, transparent process & decision making, genuine and public consultation and regular review [12].

#### Thailand

HTA institutionalization in Thailand has been viewed as a leading institutional structure globally that is conducted in a manner that incorporates evidence generation and use in policymaking, building the capacity of HTA practitioners, organizations, system infrastructure and collaborations [8]. In Thailand, the HTA mechanism is propagated into decisions of coverage and plays a prominent role in the conceptualization of the List of National Essential Medicine as well as considerations into UHC (Universal Health Coverage) benefits package scheme [8]. The guiding features of this method for institutionalization propagate values such as political will and leadership, capacity building on HTA-related disciplines, adequate resources, technical expertise, and data (8).

#### Ghana

The institutional arrangements for HTA in Ghana are in the preliminary stages. Currently, HTA is built to initiate institutionalization to inform policy making, priority setting for health interventions, reimbursement, pricing of pharmaceuticals and other health technologies [13]. HTA in Ghana was propagated by the Minister in the Ministry of Health with the inauguration of the HTA Steering Committee, Technical Working Group and Secretariat, which oversee the development of HTA under the auspices of the Ghana Ministry of Health [13]. The Ghanaian approach to HTA is spelled out in the country’s Ministry of Health’s strategic plan, whose identified purpose is strengthening the science and practice of HTA in support of evidence-based decisions to advance health provision outcomes in their nation.

#### Ethiopia

Health technology assessment (HTA) in Ethiopia exists in a fragmented manner. Among the institutional capacity, the Health Economics and Financing Analysis (HEFA) team was established within the Finance Resource Mobilization Department under the Ministry of Health [14]. The HEFA mandates and champions theapplication of evidence-based healthcare decision making in Ethiopia by organizing available evidence, costing interventions, and defining effectiveness measures of the different health programs and then supporting policymakers at the national and regional levels [14].

#### South Africa

Health care in South Africa aims to ensure that the most prominent and basic health needs of the population are met [15]. Thus, HTA was adopted as a mechanism to anchor the ideals of UHC. HTA in South Africa is both a technical and political process, involving a range of stakeholders, systems, disciplines and viewpoints [16]. The HTA process in South Africa seeks to be effectively anchored on the views and experiences of a broad range of stakeholders, across income quintiles and sectors and seeks to develop a sense of ownership [16].

### Current decision making structures for health technology adoption in Malawi

The FGD and KIIs indicated that apart from the policymakers at the Ministry of Health, there are other players in adopting new health technologies namely PMRA, Civil society, Malawi Medical council and non-governmental health care providers such as CHAM. The study participants indicated that health technology adoption in Malawi is primarily reliant on the funding source for corresponding health technology. The funding source may be in the form of:

#### Government-funded technologies

The FGD and KIIs indicated that the decision-making process for health technology adoption in the health care system in Malawi is embedded within the Ministry of Health Senior Management Team (SMT) decision-making structure. The SMT comprises the Secretary for Health, Chief of Health Services, Directors and Deputy Directors of MOH (Ministry of Health) Headquarters and Directors of the five Central Hospitals. Analysis of KIIs, FGD, and the document review results, indicate a three-stage process. First, a relevant department identifies a health technology it needs and its director presents details of the health technology of interest to a relevant Technical Working Group (TWG). A TWG membership comprises technicians from the MoH and stakeholders with appropriate expertise. When more information is needed, the TWG appoints a task force to undertake a more detailed analysis and present its findings for review and endorsement by the TWG. Following the review of the results, a TWG can recommend to the MOH SMT to adopt or not adopt the proposed health technology intervention. MOH SMThas the liberty to adopt or reject the recommendation of the TWG or refer the work back to the TWG to address any areas that SMT advises on.

The qualitative inquiry further found that to implement the adoption decision from SMT, the user department initiates a procurement request which goes through the Internal Procurement and Disposal Committee (IPDC) for scrutiny. The main task of the IPDC is to ensure that the acquisition of the technology complies with the Public Procurement Act of 2017. Based on feedback from the FGD, some of the key considerations include “*the functionality of the product or the equipment or the service in the country, availability of capital sale services and financial capacity of the bidder… […]; if given a contract [they] should be able to bring the product into the country*” (FGD Res.07_Policy Maker).

For these Government-funded technologies, the implementation of the decision by SMT is generally executed by the Department of Health Technical Support Services (DHTSS) but there are others involved such as the Diagnostics Unit, Clinical department and the Public Health Institute of Malawi. The DHTSS has three Divisions: (1) Physical Asset Management (PAM) Unit, which coordinates the procurement of technologies that focus on medical devices; (2) Pharmaceuticals Division, which coordinates the procurement of medicines and medical supplies; and (3) Laboratory Unit which coordinates the procurement of laboratory supplies and reagents. The PMRA provides regulatory oversight over medicines. However, its mandate has recently been expanded to cover diagnostics and monitor the safety and efficacy of medicines and enforce compliance with standards. A respondent for instance indicated that *“… when we see that the government would like to deal with that supplier, we write them that can you submit the dossiers, and an application to us so that we (can) assess your product*” (KII 10_Regulatory Authority).

#### Development partner-funded technologies

For these technologies, the government generally expresses the need for a technology to the donor through the coordinating department or program head. Mostly, requests for technology acquisition would be based on the priorities in the national Health Sector Strategic Plan (HSSP) or a relevant program-specific strategic plan. Two possibilities exist in the acquisition process. The first is where the partner could provide the funds to the MOH, for example, through the Health Services Joint Fund (HSJF) (a pooled fund for three Donors: UK Foreign Commonwealth & Development Office, Norway and Germany), or via a Project Implementation Unit (PIU) such as the joint PIU for GAVI and the Global Fund. In this case, the government-funded technology mechanism described above is utilized with or without adaptation depending on additional donor procedures for acquiring technologies. The second is where donor-funded projects use their internal procurement systems to acquire the technology on behalf of the Government. In this option, donor procedures will be used only after the technology has been approved by the regulatory body for the technology as explained by one FGD participant: “*But in cases of emergency, sometimes it goes directly from the head of the department to a partner such as CDC* (Centre of Disease and control), *CHAI* (Clinton Health Access Initiative), *or UNICEF* (United Nations International Children’s Emergency Fund) *to help with procurement. In this case, the procurement process happens in their system now, not the government system. And once a technology has been procured, it’s delivered to the government [which] facilitates distribution”.* (FGD Res 2_Policy maker)

#### Technologies donated in kind

There are two types of donations, fully funded donations and partially funded donations. For fully funded donations, the total cost of acquiring the health technologies is fully covered by the donor. The need for such technologies is ideally expressed by the MOH through the relevant head of the department or program. For donations that are made in direct response to an expressed need from the Government, the responsible head of the department or program institutes a task force to provide advice on the safety, efficacy and appropriateness of the technology. The Malawi Bureau of Standards and WHO standards are mostly used to inform this assessment. The task force also assesses the alignment of health technology with national health priorities and the feasibility of implementation. Based on the recommendation from the task force, a report is written to the Secretary for Health for review and possible approval that the donation is accepted. For partially donor-funded technologies, one respondent explained that “*at the point of acceptance the ministry should have appropriated the resources to support the activities that are being financed from the government side. (FGD Res 06).* Thus, given that some obligation falls on the Government, the adoption process of the technology proceeds as under government-funded technologies. The participants also reported that for donations that are not the priority for MoH, there is no distinct “HTA”.

#### Health technologies funded and distributed by the private sector

Based on the PMRA Act of 2019, for pharmaceuticals that are procured and distributed by private entities, the PMRA is responsible for their review and registration [17]. The scope of the PMRA has recently been expanded to regulate Allied Substances (Acaricides, Cosmetics, disinfectants, food Supplements, feed additives and supplements, traditional medicines), medical and surgical sundries, medical devices, reagents and condoms in addition to medicines. As a result, the PMRA regulates the registration of technologies across pharmaceuticals and medical diagnostics.

To register pharmaceuticals, a company makes an application to PMRA expressing intent to sell products on the Malawian market. PMRA then inspects the company to check its compliance with WHO Good Manufacturing Practice as required by the law and issues a compliance certificate if standards are met. The registration process involves the following steps: application for registration of the product; undertaking a bioequivalent study or laboratory proxy; presentation of the assessment report to the medicines committee of the PMRA board for endorsement; submission of the endorsed report to the PMRA board for final approval. One respondent described the process as follows: *“… there are product assessment procedures, clinical trial reviews, and pharmacovigilance safety surveillance of medicines… the (PMRA) team makes a report on every product found in retail pharmacies. They produce a report and submit it to the medicines committee of the board to consider the findings of the report*… *This committee escalates this process to the level of the board. Now the board ratifies that these products… have now been registered, they can be found on the Malawi market.” (KII 10)*.

### HTA related activities that are performed in current decision-making structures

**Fig. 1:**
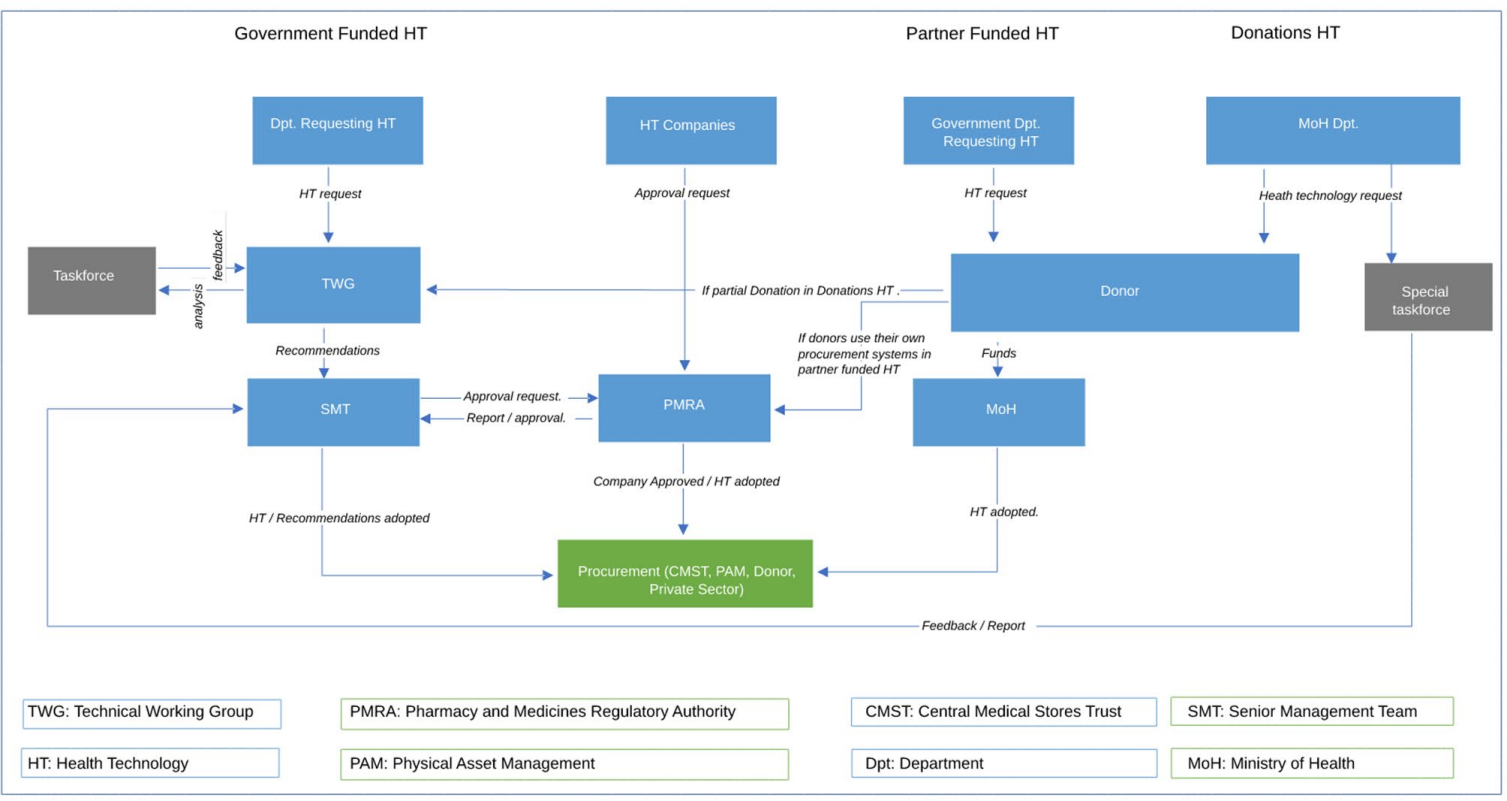
HTA Process for Pharmaceuticals and medical equipment *Source: Authors*

Based on funding streams, three distinct decision-making structures were identified, and these structures were found to execute certain functions that can inform the HTA process but no formal HTA structure exists. The informal HTA mechanisms were identified within the MOH Senior Management Team, and Technical Working Groups,while the PMRA process of product registration was pointed out by respondents as a potential candidate for the formalization of HTA. The decision pathway has been mapped out in Fig. 1.

### Values and principles guiding HTA practices in Malawi

Technology adoption for Government and donor-funded technologies is guided by existing national priorities in key policy and strategic documents. These policies and guidelines include the recently revised Essential Health Package (EHP) [18], National Health Policy, HSSP II [19], Essential Medicines List [20], Standard Treatment Guidelines [20], Malawi laboratory services and supply chain assessment [21], Public Procurement and Disposal of Assets Act − 2017 [22] and WHO guidelines. Other normative values that were mentioned by some include appropriateness, affordability, accessibility, maintenance cost and functionality of the products. An assessment of the policy documents revealed that these values are included as guiding principles without interventions and indicators for ensuring the relevant organizations build the relevant culture in their decision-making, including HTA.

The key observation provided on the use of values and principles is that they are not always adhered to leading to the misalignment of normative and actual values that are practiced as one respondent from the FGD reported: “*…although there are value sets which define the EHP process*that *informs the treatment guidelines or essential medicines list that process isn’t in existence now. So, it’s one way the values are therebut I am not sure that they are being translated into the process thatinforms what’s procured.*” (FGD Res.005).

### Strengths and challenges of partial HTA mechanisms in current decision-making structures

To determine whether institutionalizing HTA is feasible, it is important to understand the strengths, weaknesses, opportunities and threats posed within each of the informal HTA mechanisms that have been identified within the decision-making structures. The respondents reported various challenges affecting the current decision-making processes of assessing health technologies for adoption in the Malawian health system. These include limited use of evidence, lack of a standardized framework, political interference, lack of resources, pressure from donors, difficulties in implementing cost-effective interventions due to conflicting policies, and lack of transparency and accountability. Table 1 below summarizes the Strengths, Weaknesses, Opportunities and Threats of informal HTA Mechanisms in Malawi.

**Table 1:** Strengths, Weaknesses, Opportunities and Threats of informal HTA Mechanisms

	MOH SMT/TWG	PMRA
**Strengths**	•Greater alignment of policy questions to health system goals •Senior management composition could mitigate any risks in decision-making arising from a weak evidence base including cost-effectiveness •The multi-stakeholder composition of TWG members provides scope for transparency and accountability.	•Available capacity for handling issues of efficacy and effectiveness •Has legal Mandate-Act of Parliament •Has a significant level of independence •Is already funded by the public, donor and private funding streams •Its mandate already stretches across government, donor, and privately funded technologies •Minimal political interference as oversight is through a Board of Trustees •Has robust internal research capacity for pharmacovigilance
**Weaknesses**	•SMT can be pressured by donors •Political economy interests of top decision makers could prevail over value and evidenced-based decision making •Weak institutional mechanism for evidence synthesis	•Its statutory corporation status could limit the scope for its execution in the event of interference by MOH and other central government institutions •It does not have the capacity for cost-effectiveness analysis •Poor coordination with health technology procurement institutions •Lack of WHO prequalification of the laboratory limits the assessment of some pharmaceuticals •Globally, it is not a standard practice that HTA is placed in the medicines board.
**Opportunities**	•It is an existing decision-making structure of MOH •Any identified institutional and funding gaps can be reasonably addressed •TWG structure is acceptable by donors and wider stakeholders •TWG structure provides access to top researchers who can contribute to the evidence process at no cost to the Government	•New regulations extend its mandate to cover all health technologies rather than medicines only •Has the trust of all stakeholders in health technology
**Threats**	•Political interference •Could be influenced by political economy considerations	•Funding for HTA could be diverted for other regulatory activities •Staff establishment restrictions could limit the potential for in-house capacity in health economics

### Institutionalizing HTA in Malawi: demand and options for institutionalization

For the institutionalization of HTA, there must be demand for it. This was ascertained by asking FGD and KII informants whether or not HTA would improve decision-making in the health sector. From this question, we further inquired what form HTA institutionalization could take. Based on interviewee responses, the need for HTA was extensively highlighted and deemed necessary. The participants expressed that an institutionalized HTA would fill a regulatory gap for HTA adoption across multiple channels. The participants further iterated that an institutionalized HTA will facilitate cost-effectiveness considerations in decision-making for both medical devices and medicines including donations. A participant suggested that HTA *“will serve as a guiding tool to make sure that there is competition on the market” and ensure objectivity when … “selecting these manufacturers or suppliers”* (KII_06). The participants further proffered that, the institutionalization of HTA would provide coordination among stakeholders involved and improve relationships across institutions. Additionally, institutionalizing HTA in Malawi would improve decision-making regarding technology adoption, and contribute to efficiency, equity and transparency in health financing, thereby leading to improved service delivery and results.

Several options for institutionalizing HTA in Malawi were proposed by participants. Based on the functions already undertaken by PMRA, most respondents who were conversant with HTA processes or practices suggested that efforts towards institutionalizing HTA should constitute empowering the PMRA. PMRA has a new regulatory framework that was drafted and has *“expanded the scope to include all aspects of technology such as medical devices and traditional medicine currently excluded in the PMRA Act”.* (KII_06). Therefore, the respondents suggested exploring the feasibility of capacitating PMRA to perform HTA functions. Another suggested option for institutionalization was that HTA should be established as a standalone institution independent of MoH. A third option that respondents proposed was setting up an HTA body within an appropriate academic institution considering the strong research culture that exists in academia, upon which the evidence base for HTA could be harnessed. A fourth option was establishing a unit specializing in HTA within the department of planning in the MoH, PMRA or possibly under the Public Health Institute of Malawi (PHIM) in the department of research. The respondents highlighted that this would ensure that HTA is ***“****housed within the ministry as this is where the majority of the decisions are made in regards to health technology*” *(KII_08)*. A final option mentioned was to set up HTA as a *“committee and eventually develop that into a full department within one of the government functions” (KII_08)*, once the value of HTA has been established.

The participants proposed multiple ways in which HTA could be funded in Malawi. These options included but were not limited to funding from the government and international development partners. Another proposition was to have a subscription-based system for all partners that would have a role in the HTAframework.

## Discussion

This section presents a discussion of the findings of the study and recommendations of plausible ways in which HTA can be institutionalized in a low-income setting such as Malawi given the pertinent challenges that exist in its health sector. After a thorough understanding of the dimensions that guide decision-making in health technology prioritization and adoption in Malawi, the need for an institutionalized HTA mechanism was found to be acceptable and implementation was deemed feasible. In a lower resource setting such as Malawi, HTA has the potential to play a significant role in the incorporation and utilization of health technology processes, by contributing to the equitable provision and access to healthcare services, more efficiency in the allocation of resources, better effectiveness and quality of services, and stronger financial sustainability of the healthcare system [7, 23].

### The nature of decision making and HTA in Malawi

The study foundthat there is no institutionalized HTA mechanism in Malawi though some partial HTA processes exist at different points in the health technology adoption process. The partial HTA mechanisms exist primarily in the MOH Senior Management Team, Technical Working Groups, and the PMRA which were all found to have varied roles of effectiveness. It was established that Malawi relies primarily on committee-based decision-making processes.Because they do not utilize consistent methods across channels, they are prone and face difficulty in implementing cost-effective interventions, political interference, pressure from donors, limited and unsystematic use of evidence, and partial adequacy of capacity at PMRA. These findings are consistent with experiences in other LICswhere either HTA does not exist at all or exist as partial processes at different phases of the health technology adoption or prioritization process [3, 23].

Even though a formal HTA program might not be in place in a given country, decision-making about the adoption of new technologies may be part of the operational routine of health authorities and health service providers [24]. In this case, decision-making is frequently based on unilateral industry information, particular interests of individuals, or ‘gut feelings’ which may prove futile, unreliable and even disruptive to the decision-making process [4, 24]. This is evident in the case of Malawi where decision-making on health technology prioritization and adoption is marred by limited or inadequate evidence use, political interference, value misalignment between the EHP and other corresponding policies such as the Essential Medicines List and The Standard Treatment Guidelines. To rectify such challenges, health systems shift from “sporadic decision making” to decision-making processes that follow systematic modern principles such as Evidence Based Medicine (EBM), cost effectiveness and patient centered services that have the capacity to translate into the establishment of an institutionalized HTA [24].

### Moving from informal assessments to formal, harmonized and institutionalized HTA

In general, HTA has its prominence in high- and middle-income countries. The major focus in the world has been placed on England and Wales which are deemed a pioneer institutions where NICE conducts appraisals using the evidence coming from HTA, in a process that leads to guidance [25]; a term they refer to as HTA beyond decision making [6]. Similarly, in middle - high-income countries such as Thailand, HTA has been formally integrated into coverage decisions, including in the development of the National List of Essential Medicines and the UHC Scheme benefits package [3]. Drawing lessons from NICE and HITAP (Health Intervention and Technology Assessment Program) and how they are expanding their mandate on HTA to other low-income countries such as Myanmar, The Philippines, Vietnam and Columbia could also provide firm direction on a potential pathway Malawi can take in institutionalizing HTA [6].

Literature shows that moving to a formalized and systematic HTA program requires a solid commitment from governmental authorities and a designated and motivated team of professionals that take charge of the HTA development plan [4]. Siegfried et al. (2017), reports that there is no ‘one-size-fits-all’ for the delivery of HTA, rather HTA can be institutionalized in varying degrees according to each relative context [16]. A good approach to HTA institutionalization can be to design a system that caters to the unique policy needs of that context, becoming an institution that works withexisting funding structures and boosts the nature and availability of evidence and existing approaches to decision-making [26]. And whether the HTA is successful or not can be established as the extent to which it contributes to defined policy objectives such as achieving value for money and improving health outcomes, and addressing inequalities and access to health technologies [16, 27].

### Institutionalizing HTA in a LIC: the case of Malawi

To establish a formalized HTA institution in a LIC such as Malawi there is a need to establish a mandate among preliminary stakeholders that would warrant a critical role in the establishment of the institution. These stakeholders would be the primary parties responsible for decision-making (policy brokers) and relevant parties involved in the prioritization and adoption of health technologies in the country. In Malawi, these stakeholders could potentially be the MoH, Central as well as District Hospitals, Civil Society, CMST, Development/ Donor Partners, CHAM, PHIM and regulatory authorities such as the Medical Council and PMRA. Secondly, there would be a need for the development of a legal framework, establishment of institutional arrangements, definition of procedural aspects of assessment and appraisal and a proposed monitoring and evaluation component of the HTA mechanism [26]. Importantly, some reforms would be required such as having the Planning and Policy Directorate of the MOH decide whether to continue with the current practice of committee-based decision-making processes or shift towards a hybrid, or a formal institutionalized HTA.

As others have noted, the institutionalization of HTA requires the identification of appropriate processes, adapted to the country’s context regarding available capacities [28]. In the case of Malawi, given the partial HTA processes that exist, a major premise of institutionalizing HTA could border on growing from or expanding on prioritization and adoption processes (decision-making structures) that currently exist within relevant institutions explored within the study. These institutions could be fine-tuned as a basis for building a comprehensive HTA institution with distinct as well as complementary steps and processes that aid cost-effective decision-making. These processes could be, (a) developing the HTA capacity of PMRA to act as an HTA agency operating under regulation, (b) Strengthening the capacity of MOH Senior Management and District Councils in HTA agenda setting as a phased approach towards institutionalizing HTA, (c) Establishing Cost-Effectiveness Analysis capacity of MOH and TWGs to aid evidence generation and synthesis, (d) Developing capacity to conduct cost-effectiveness analysis and evidence generation within academia, as well as (e) building sustainable financing for HTA.

#### Developing HTA functions alongside the regulatory capacity of PMRA

As found in this study, the PMRA is mandated with regulatory oversight of the registration of health technologies but not the authority over what technologies to fund and not to fund. The main attraction of an embedded HTA function within the PMRA would be that it already has a B oard and is regulated by a Parliamentary Act. This could provide significant independence of the HTA entity within PMRA. Limitations with this approach however is that PMRA exists to execute a regulatory function and extending this to HTA could result in conflict of mandates.

### Strengthening capacity of MOH senior management and district councils in HTA agenda setting

Consistent with what others have reported [29], we recognize that the lack of human resources with requisite skills is a challenge to the institutionalization of HTAs. For HTA agenda setting, the roles of MOH and district councils will thus require strengthening regardless of the form of HTA arrangements since they are the fund holders of Government money. This capacity building could extend to developing competencies of these institutions as gate-keepers of donor-procured or donated technologies so that principles of cost-effectiveness are also extended to productsprocured on behalf of Government facilities outside of Government arrangements. Nevertheless, this will require undertaking a needs assessment and developing responsive capacity in line with the overall HTA architecture. This will then inform both the national level institutional setup consistent with optimal HTA processes.

#### Establishing cost-effectiveness analysis capacity of MOH and TWGs

Within MOH institutional arrangements, there would be a need to develop internal capacity to support the agenda setting and funding decision processes by strengthening the capacity of the Health Financing Division (HFD). Presently these functions are diffused between the budget division and the policy development division. The mandate of the HFD is to identify and facilitate cost-effectiveness assessments of both government and donor-funded interventions including technologies. Also, the HFD is the custodian of Memorandums of Understanding (MOUs) between the Government and its development partners in the health sector. This places the HFD as an ideal link between HTA and the implementation scale-up of MOH and donor-funded technologies. The health financing TWG, to which the HFD is the secretariat, provides a technical advisory role to the MOH Senior management. This could provide a layer between HTA evidence demand and MOH decision-making. This suggests that in the short to medium term, a Unit could be established within the HFD to act as secretariate and coordinate all relevant HTA capacities. Critically, the success of the HTA function will depend on effective coordination, internally, among the HTA unit and the rest of stakeholders, notably the Public Health Institute which houses the Research Department, other key stakeholders relevant for HTA decision making, and research collaborations. Overtime, the Unit could graduate into an independent HTA body.

#### Developing the capacity to conduct effectiveness analysis and evidence generation within academia

Also, there is an option to explore the Government facilitated and funded the Health Economics and Policy Unit (HEPU) at the Kamuzu University of Health Sciences. The Government can leverage this collaboration by tasking the HEPU with policy analysis function in support of the HTA capacity gaps within the MOH and HTA assigned structures such as PMRA. This specialized capacity within HEPU can be developed either as “offshore” HTA capacity fully aligned to MOH and executing HTA evidence and knowledge management function in a sub-contractor capacity. This option will allow MOH to focus on agenda setting and decision making in line with its policy oversight mandate while HEPU and other academic institutions with relevant HTA capacity can provide the evidence support. At the time of this study, the Government had already commissioned various value for money assessments to HEPU. Over time, this deliverable could be strengthened and institutionalized as a robust HTA evidence support structure.

### Building sustainable financing for HTA

HTA bodies may be eligible to receive public funding and be established by ministries of health (at national, provincial or regional level), receive a mix of private and public funding, be independent of governments, be situated within academia or initiated by organizations of health professionals [28]. For effective HTA, sustainable and ear-marked funding for the HTA mechanisms should be explored and sustained over time. For academic institutions, this could be supplemented by research grants that support cost-effective analysis. Finally, fundingcan come from submissions for privately funded requests from companies through the manufacturers. Sufficient investment funds should be made available to train professionals in HTA work. Funding for the recurrent operational costs of the established HTA structure should be identified and secured on along-term basis. HTA work is no longer done in national isolation. The national HTA concept should include an international network strategy right from the beginning [24].

## Conclusion

In Malawi, HTA is feasible and acceptable in order to promote equitable, efficient, fair and transparent resource allocation and prioritization. The studyrevealed three salient HTA structures for decision- making:committee-based systems, a hybrid of committee based and partial HTA institutional mechanisms and a fully functional institutionalized HTA. Malawi has a strong demand and need for an institutionalized HTA and strengthening of the capacity of the Cost Effective Analysis. Lastly, the evidencedemonstrates the need for country-specific HTA assessments before creating new HTA institutions in order to customize HTA frameworksto political and managerial contexts.

### Supplementary

Focus Group Interview Guide and the Key Informant Interview Guide and Consent Form are attached with submission.

## Electronic supplementary material

Below is the link to the electronic supplementary material.

Key Informant Interview Guide

Focus Group Discussion Guide

## Data Availability

The datasets used and/or analyzed during the current study are available from the corresponding author on reasonable request.

## List of Abbreviations

AHRQ: Agency for Healthcare Research and Quality
CDC: Centre of Disease and Control
CEA: Cost Effectiveness Analysis
CHAI: Clinton Health Access Initiative
CHAM: Christian Health Association of Malawi
CMST: Central Medical Stores Trust
COMREC: College of Medicine Research Committee
CONITEC: National Committee for Health Technology Incorporation
DHTSS: Department of Health Technical Support Services
EHP: Essential Health Package
EUnetHTA: European Network for Health Technology Assessment
FGD: Focus Group Discussion
HAS: Haute Autorité de Santé
HEFA: Health Economics and Financing Analysis
HEPU: Health Economics and Policy Unit
HF: Health Financing
HFD: Health Financing Division
HSJF: Health Services Joint Fund
HSSP II: Health Service Strategic Plan II
HTA: Health Technology Assessment
HTAI: Health Technology Assessment International
HITAP: Health Intervention and Technology Assessment Program
IETS: Institute of Health Technology Assessment
INAHTA: International Network of Agencies for HTA
IQWiG: Institute for Quality and Efficiency in Health Care
KuHes: Kamuzu University of Health Sciences
KII: Key Informant Interviews
LICs: Low-income countries
MOU: Memorandum of Understanding
MOH: Ministry of Health
NICE: National Institute ofHealth and Care Excellence
PAM: Physical Asset Management
PIU: Project Implementation Unit
PHIM: Public Health Institute of Malawi
PMRA: Pharmacy and Medicines Regulatory Authority
SMT: Senior Management Team
TWG: Technical Working Group
UHC: Universal Health Coverage
UNICEF: United Nations International Children’s Emergency Fund
WHO: World Health Organization
