# Supplementary material for: A qualitative study on the feasibility and acceptability of institutionalizing health technology assessment in Malawi
Source: BMC Health Serv Res. 2023 Apr 11;23:353. doi: 10.1186/s12913-023-09276-z (PMC10088659; doi:10.1186/s12913-023-09276-z)
Supplement: Supplementary file 1 — Key Informant Interview Guide [file 12913_2023_9276_MOESM1_ESM.docx]

**Key Informant Interview Guide**

**Health Technology Assessment & Value and Evidence Based Decision Making**

| Start time: __________________________ End time: __________________________________Participant ID: _______________________ Position: ___________________________________Institution______________________________ District _________________ **Facilitator’s Name:** ____________________________________  **Date:** \|__\|__\|/\|__\|__\|/__ **__**  ***day month Year*** |
| --- |

##

| **Instructions to Facilitators** |
| --- |
| **Preparing for the interview**  Greetings and self-introduction: Hello, my name is _____________________ and I am working on behalf of University of Malawi College of Medicine (Health Economics and Policy Unit) and University of York. We want to get some information regarding the decision making processes on identification, prioritization and adoption of health technologies; wherein Health Technologies are defined as the application of organized knowledge and skills in the form of devices, medicines, vaccines, procedures and systems developed to solve a health problem and improve quality of lives. This research will help to provide a detailed understanding of the context in which resource allocation decisions are made and used in health sector. We will be asking questions, taking notes and recording your responses so that we can capture everything clearly. Our discussion will last for about 30-40 minutes. Please feel free and open to share your views in this discussion, and to ask questions where you don’t understand, or you want clarification. Your participation in this study is voluntary and thus it is up to you to accept or refuse. If you choose not to participate, you will not be affected in any way.  Do you consent for us to continue with the interview and/or audio recorded?  (obtain consent through verbal confirmation or written) |

**Section 1: Identification and Prioritisation of HT- to be answered by pharmaceuticals representatives, CHAM and Hospital directors.**

**A: Decision Making Process**

1. Tell us about your role within the ministry/department/hospital/organization.
2. How are decisions about adoption and exclusion of Technologies and medicinal/pharmaceuticals procurement made in your/this institution? Probe:
   - 1. Who makes decisions relating to introduction or removing of technologies and drugs. Are there existing structures? Who funds these structures?
     2. Values, evidence, and policies aiding decision making of technology and medicinal acquisition?
     3. Who makes the final decision?
     4. How do you ensure that the process is transparent, accountable and responsible?
3. To what extent are you involved in processes relating to adoption of pharmaceuticals and other health technologies?
4. What are the challenges with the current decision making process in regard to the adoption of pharmaceuticals, vaccines, devices etc.?
5. Do people receive pressure from manufacturers, patient groups or proposals by the donor agency to procure a particular type of technology? If yes, please explain what kind of pressure?
6. How do you think this process can be improved?

**B: Funding**

1. Where does the funding to procure pharmaceuticals come from?
   - 1. If funding is through donors, how do the people make the decision to accept the donation?
2. Given the scarcity of resources, how do you ensure that the money is invested in the best way possible? (probe about opportunity cost)
3. What are the challenges faced during the process of procuring the pharmaceuticals?
4. In your opinion how would the process of procuring pharmaceuticals be improved?

**C: Values**

1. Are there standard criteria guiding decision-making for inclusion and exclusion of drugs and technologies? If yes, what are they? Or are decisions only based on feedback from clinicians?
2. Are there any international recommendations and guidelines for the provision of health technologies? If yes, what are they?
3. What values guide the decision-making process?
   - 1. Cost-effectiveness, affordability, safety,
4. What values are considered most important when making a decision for health technologies?

**Section 2: HTA Feasibility and Acceptability- to be responded by all Participants**

1. How do you understand Health Technology assessment?
2. Do you think Malawi needs HTA?
3. If HTA is formally adopted, what would make it possible to easily implement it (strengths)?
4. What would be the challenge?
5. es of implementing HTA in Malawi (weaknesses)?
6. What would be the advantages of having HTA in Malawi (opportunities)?
7. What would be the risk of having HTA in Malawi (threats)?
8. In your view do you think HTA is a priority for the Malawi? Why?
9. At the moment decisions about procurement of drugs, devices and vaccines are made by various directorates in the ministry of health through the involvement of technical working groups, senior management and the secretary for health.
10. What do you think of the idea of formally establishing an institution to conduct Health Technology assessment in Malawi? (Probe: relevance and benefits)
11. What challenges would this HTA institution experience?
12. What opportunities and threats would be there for the HTA institution?
13. What do you think could be the best structure/form for HTA in Malawi (options: committee, institution, department within the MOH, or an independent statutory, a statutory board under government body like the Malawi Pharmacy medicines and Poisons Board etc.)? Probe: Based on your opinion where should the HTA institution be established?
14. Which organizations would comprise relevant stakeholders for the HTA institution of the form suggested above?
15. Who could be the potential funders for an HTA institution in Malawi?

**Section 3: Quality Control and Accountability of HT - To be answered by PAM, Hospital Directors, Administrators and CHAM.**

1. Describe the process of quality check of a new technology
   1. Who is responsible for quality control, auditing and monitoring the implementation of Health Technologies? Why?
   2. Who are they (those responsible for auditing, quality control and monitoring) accountable to? Why?
2. Who is responsible for the maintenance of the equipment? Government or user?
3. Who covers the maintenance costs?
4. How are health technologies that are no longer operational disposed of?
5. What are the strengths of the current quality control, auditing, monitoring process?
6. What are the challenges with the current quality control, auditing, monitoring process?
7. What would you recommend to be done to improve the process of auditing, quality control and monitoring?

**Section 4: Knowledge and use of normative values and establishing actual values on the ground. To be answered by hospital directors and admins**

1. What policies or guidelines do people apply in the line of their duties in the ministry of health and hospitals?
2. Are people aware of the policies and guidelines that guide the decision making and operations within your institution? What are they? (e.g. Policy documents such as EHP, HSSP II and National Health Policy have some values which are supposed to be followed when implementing activities in the ministry of health and hospitals, i.e. transparency, accountability, effectiveness, efficiency, quality and demonstrable value for money.)
   1. Are the policies and guidelines applied in the decisions that are made and in the operations? (if not, why not? If yes, why?)
   2. Are they needed/necessary? Why?
   3. What values do you deem as important? Why?
   4. Which values do people apply the most in their line of work?
   5. What do the people say about the policies and guidelines?
3. What values are used when making decisions? (probe: to what extent are the values used?)
4. What other values do you think would help improve decision making process?
5. Of the values which are currently in use which ones would you suggest to be dropped?
6. What are the barriers preventing the application of such values? (probe: of the values not applied what are the reasons)
7. What aspects of your department would you want to change when it comes to decision making regarding acquisition of medical equipment, vaccines, medicines etc.?
