# Supplementary material for: A qualitative study on the feasibility and acceptability of institutionalizing health technology assessment in Malawi
Source: BMC Health Serv Res. 2023 Apr 11;23:353. doi: 10.1186/s12913-023-09276-z (PMC10088659; doi:10.1186/s12913-023-09276-z)
Supplement: Supplementary file 2 — Focus Group Discussion Guide [file 12913_2023_9276_MOESM2_ESM.docx]

*Feasibility and Acceptability of Establishing Health Technology Assessment in a Low Income Country Setting: The Case of Malawi*

# Focus Group Discussion Guide

*18.11.20*

**Purpose**

The purpose of this focus group discussion is to gather data on health technologies in Malawi. The discussion seeks to explore and establish the decision making processes centred on the identification, prioritization and adoption of health technologies; wherein Health Technologies are defined as the application of organized knowledge and skills in the form of devices, medicines, vaccines, procedures and systems developed to solve a health problem and improve quality of lives.

The discussion also seeks to further definitively explore the values and value sets that substantiate and govern the identification, prioritization and adoption of health technologies in Malawi, were we seek to understand how values such as evidence, cost – effectiveness, transparency, ethical considerations, acceptability, efficiency and efficacy are considered throughout these processes.

# Interview Guide

*Step 1: Identification and Prioritization*

We want to understand how technologies are introduced in Malawi. Having divided the adoption of health technologies, drugs and medical devices into four respective guise – i.e. donations, pharmaceuticals, diagnostic equipment and vaccines); we wish to explore the identification, prioritization and adoption of Health Technologies in the country.

*Question 1*

With the aid of the examples highlighted above, could participants highlight the process for introducing medicines (pharmaceuticals), diagnostics/ medical equipment’s, and possibly vaccines through;

- Process (pharmaceuticals, medical equipment & vaccines) procured through/ under government funds
- Process (pharmaceuticals, medical equipment & vaccines) procured through donor funds
- Pharmaceuticals/ diagnostics donated in kind.

*Question 2*

- Who are the main actors in the decision-making processes for identification, prioritization and adoption of health technology in Malawi? *(Probe: What are their roles?)*
- Is there a generic decision-making organogram as follows? Is the structure accurate? *Confirm organogram and ask the following questions*
- *Who constitutes the task force – what is the role of the task force? Who is in the task force?*
- *What are the technical working groups? What are their roles? Who is in the TWGs?*
- *Who constitutes the senior management teams? What is their role?*

*Question 3*

- Apart from the EHP, HSSP II and the National Health Policy are there any other policies guidelines that aid decision-making practices in Malawi. *(Probe: If yes, what are they? (Can also be established through – Desk Review – Key informant interviews))*

***Step 2: Value Sets in Prioritization***

*Question 4*

- What values guide the prioritization process? Are there any relevant criteria for adoption? (*Probe: Value for money (i.e. Cost-effectiveness), Transparency, Regulations, ethical considerations, political will, efficacy)*

*Question 5*

- Do you use evidence? What type of evidence is needed and where is it generated? Is there a process of evidence synthesis? Are there any mechanisms to rank or score the tangibility of the evidence?

*Question 6*

- What are the current bottlenecks and major challenges within decision-making structures?

*Step 3: Funding*

*Question 7*

- *How are the new technologies financed, if adopted?*
- How does the source of funding for a technology affect adoption decision?
- Given the scarcity of resources, how do you ensure that the money is invested in the best way possible? (probe: about opportunity cost)

*Step 4: Adoption*

*Question 8*

- What type of values and framework is used to decide on adoption of technologies and for whom?
- Is all criterion of linked to evidence and analysis; are criteria generic or do they change on a case-by-case basis?
- How can the transparency of process be validated or ensured?
